# It is lonely at the front: contrasting evolutionary trajectories in male and female invaders

**DOI:** 10.1098/rsos.160687

**Published:** 2016-12-21

**Authors:** Cameron M. Hudson, Gregory P. Brown, Richard Shine

**Affiliations:** School of Life and Environmental Sciences A08, University of Sydney, Sydney, New South Wales 2006, Australia

**Keywords:** *Bufo marinus*, *Rhinella marina*, evolution, invasive species, morphology, sexual dimorphism

## Abstract

Invasive species often exhibit rapid evolutionary changes, and can provide powerful insights into the selective forces shaping phenotypic traits that influence dispersal rates and/or sexual interactions. Invasions also may modify sexual dimorphism. We measured relative lengths of forelimbs and hindlimbs of more than 3000 field-caught adult cane toads (*Rhinella marina*) from 67 sites in Hawai'i and Australia (1–80 years post-colonization), along with 489 captive-bred individuals from multiple Australian sites raised in a ‘common garden’ (to examine heritability and reduce environmental influences on morphology). As cane toads spread from east to west across Australia, the ancestral condition (long limbs, especially in males) was modified. Limb length relative to body size was first reduced (perhaps owing to natural selection on locomotor ability), but then increased again (perhaps owing to spatial sorting) in the invasion vanguard. In contrast, the sex disparity in relative limb length has progressively decreased during the toads' Australian invasion. Offspring reared in a common environment exhibited similar geographical divergences in morphology as did wild-caught animals, suggesting a genetic basis to the changes. Limb dimensions showed significant heritability (2–17%), consistent with the possibility of an evolved response. Cane toad populations thus have undergone a major shift in sexual dimorphism in relative limb lengths during their brief (81 years) spread through tropical Australia.

## 1. Introduction

Invasive species offer unparalleled opportunities to explore the process of rapid evolutionary change [1]. As an alien species spreads through previously uncolonized territory, it is likely to encounter novel selective forces (both biotic and abiotic); and the process of continuous range expansion introduces an additional set of evolutionary processes (e.g. genetic drift, mutation surfing, spatial sorting [2]). As a result, alien taxa often accumulate substantial phenotypic changes, at a timescale much quicker than usually envisaged for evolutionary change [1,3,4]. One interesting subset of traits that might be expected to evolve during a biological invasion involves sexually dimorphic characteristics. Morphological disparities between conspecific males and females take many forms [5], but evolutionary theory suggests that natural selection during an invasion might act most forcefully on traits that affect dispersal rate and/or reproductive characteristics [3].

Dispersal rate is a key feature of invasion biology, and an extensive literature suggests that dispersal rate typically evolves upwards during an invasion [6–10]. As a result, individuals in the invasion vanguard tend to exhibit dispersal-enhancing features (such as seeds that drift further on the wind, larger feet, wings or flight muscles [3,11–15]) relative to conspecifics in the range-core. Alleles that code for fast-dispersal morphological traits may accumulate in the invasion vanguard because of spatial sorting (successive generations of interbreeding between the fastest dispersers [2]) or natural selection (reflecting fitness benefits to unusually fast-moving individuals [16]). However, what happens to this acceleration if males and females within a population differ in traits (body size, limb dimensions, wing size, frequency of winged morph, etc.) that influence dispersal rate? If one sex is intrinsically faster than the other, individuals of that sex may be under intense counter-selection against more rapid dispersal, because they would encounter no potential mates at the expanding range-edge [17]. Thus, we might expect to see an evolutionary reduction in the magnitude of sexual dimorphism in traits that enhance dispersal rate.

Sexual selection plays a critical role in the evolution of sexually dimorphic traits, reflecting the way that specific morphological features affect individual reproductive success [5,18]. As a result, geographical differences in mating systems within widespread species often are associated with variation in patterns of sexual dimorphism [19,20]. By definition, population density is low at an invasion front (density is zero immediately in advance of the front) and, hence, individuals in the invasion vanguard may face very different rates of encounter with mates and consexual rivals than would be the case in the range-core [21]. That disparity would be exacerbated by any shifts in the operational sex ratio between the range-edge and range-core [21]. In circumstances where mates are rare, individual fitness will depend on traits that promote survival and longevity rather than success in mate competition [22–25]. In contrast, high population density can increase intrasexual competition and result in the evolution of secondary sexual characters such as ornaments, weaponry or sexual size dimorphism [5,18,26,27]. This is especially true for the sex in which reproductive success is limited by access to mates (typically males [21]), and stems from the disparity in genetic interests between the sexes (‘sexual conflict’ [18,28]). Thus, both the ‘dispersal rate’ and ‘sexual selection’ hypotheses predict the evolution of reduced sexual dimorphism at an expanding range-edge.

To test this prediction, we gathered data on cane toads (*Rhinella marina;* formerly *Bufo marinus*) from 67 populations across the species' invaded range in Hawai'i and tropical Australia. Importantly, dates of introduction or arrival at each of these sites are well documented, allowing us to explore shifts in sexual dimorphism as a function of time since colonization (TSC). Densities of invasive cane toad populations vary dramatically with TSC [29]. Comparisons between range-edge and range-core toad populations within tropical Australia have demonstrated substantial shifts in morphology [30,31], locomotion [32,33], dispersal ability [2,8,9,34–37], hydric and thermal tolerance [38,39], immune function [40], life-history traits [41], reproductive frequency [42], incidence of spinal arthritis [43] and larval plasticity [44]. Thus, cane toads are a plausible candidate species for examining invasion-associated shifts in sexual dimorphism.

We chose to focus on length of forelimbs and hindlimbs relative to the toad's snout–vent (body) length (SVL). As well as being straightforward to measure accurately, limb dimensions have been reported to influence mating success of male cane toads [45,46], and toads with longer hindlimbs travel further distances daily when radio-tracked [30]. Thus, relative limb length is a likely target of natural selection and spatial sorting as well as sexual selection. Additionally, our preliminary analyses revealed significant sexual dimorphism in this trait: male cane toads have longer limbs (relative to body length) than do conspecific females, a pattern that is also widespread in other amphibians [47].

Simply documenting geographical variation in a trait (or dimorphism in that trait) does not establish any evolutionary causation, however. Anuran amphibians (including cane toads) exhibit high levels of developmental plasticity. The conditions in a water body (temperature, food supply, competitor density, predator cues, etc.) can significantly affect the morphological traits of toads that metamorphose from that site [48,49]. In at least some anurans, rearing conditions of tadpoles can influence relative limb lengths of metamorphs [50–53]. To sustain an explanation couched in terms of evolution rather than developmental plasticity, we need to breed toads in captivity, and raise their offspring to maturity in standard conditions. Unless offspring resemble their parents in relative limb length, and geographical divergences in morphology among wild-caught animals are also seen in their offspring even after the latter are raised in standardized conditions, we cannot attribute divergences among populations to evolutionary forces rather than to plasticity. Quantifying heritability also allows us to determine if morphological traits are likely to be able to respond to selection. We thus also conducted a breeding study.

## 2. Material and methods

### 2.1. Capture of specimens and sites of collection

From August 2013 to March 2016, we collected adult cane toads from sites along a transect in tropical Australia (total *N* = 2076; Queensland *N* = 467, Northern Territory *N* = 802, Western Australia *N* = 807). We also collected toads from three Hawaiian islands (*N* = 1018; Hawai'i *N* = 529, O'ahu *N* = 333, Maui *N* = 156) between January and July of 2015. Toads were captured by hand, and we used vernier callipers (±0.1 mm) to measure the SVL and limb lengths of each toad. The hand, radioulna and humerus were measured for the forelimb (arm), whereas the femur, tibiofibula and foot were measured for the hindlimb (leg). Values for each component of a limb were added together to obtain measures of total forelimb length and total hindlimb length. Toads were sexed by examining external morphological characteristics (e.g. males possess nuptial pads on the thumbs, rugose dorsal skin and yellow coloration) and vocalizations (e.g. males observed calling, or producing release calls upon handling). Most individuals greater than 90 mm in SVL are sexually mature [54].

### 2.2. Common garden offspring

We collected a subgroup of adult Australian cane toads (approx. 25 males and 25 females per population) from the two extremes of the invaded range to conduct a ‘common garden’ breeding experiment. These toads were collected from three long-established populations in northeastern Queensland (more than 70 years since colonization; Townsville, Innisfail, Tully) and four recently colonized sites in northern Western Australia (less than 3 years since colonization; El Questro, Purnululu, Wyndham, Oombulgurri). Using protocols outlined by [55], we induced breeding pairs to spawn by injection of leuprorelin acetate (Lucrin; Abbott Australasia, Botany, NSW) using 1 ml of Lucrin diluted 1 : 20 with saline and raised the resulting progeny in captivity at our field station in the Northern Territory (12°37′ S, 131°18′ E). All dams and sires were bred only once in this study, thus F_1_ individuals from each clutch were full-siblings. Once metamorphic toads attained SVLs more than 20 mm, we toe-clipped them for identification and moved them into outdoor enclosures in groups of 30 (with mixed parental origins). As F_1_ toads grew, they were split into smaller groups (approx. 10 by adulthood) to reduce competition for food and space, and avoid cannibalism. From this common garden study, we obtained data on 489 captive-raised offspring (287 Queensland, 202 Western Australia) from 31 egg clutches (16 Queensland, 15 Western Australia). We measured the same traits on these offspring as we did on wild toads. Offspring were measured at approximately 2, 8, 14 and 17 months of age to quantify changes in skeletal morphology with growth and maturity. By their fourth measurement, 184 individuals had reached maturity and could be sexed based on the same criteria as used for wild-caught toads (see above).

### 2.3. Statistical analyses

We used multiple regressions to assess the effects of sex and TSC (in categories of 0–10 years, 11–20 years, 41–50, 51–60, 71–80 years) on relative arm and leg lengths of wild toads. The SVL was included as a covariate in the models to control for body size. For graphical purposes, we calculated % limb length by dividing arm and leg measures by SVL. We calculated the difference in mean values of % limb lengths (for arms and legs separately) of males and females as an index of sexual dimorphism. To estimate the repeatability of our measurements of toad limbs, on one occasion, we took triplicate measures from five individuals. We used the R package rptR [56] to calculate repeatability of measures of each limb component.

We used linear-mixed models to determine whether the relative length of limbs (and their components) of male and female toads raised in a common environment were affected by their parents' location of origin (long-established populations in QLD versus invasive populations in WA). State and sex and their interaction were included as fixed effects in the models, along with SVL to control for body size. Individual ID (nested within clutch and state) and clutch (nested within state) were included as random effects in the models. This analysis was conducted on the final measurements made on the 184 individuals that were mature at the end of the study (76 F, 108 M).

To compare patterns of relative limb length of toads raised under common garden conditions to those exhibited by wild-caught toads from QLD and WA, we used a subset of the data on wild toads (limited to individuals from those two regions). We used multiple regression with state and sex and their interaction as independent variables.

As an additional, more formal test of the effect of rearing environment on locational differences in morphological sexual size dimorphism (SSD), we performed multiple regression combining data from wild-caught and common garden toads. The model included full factorial interactions among sex, state (WA versus QLD) and source (wild versus common garden). SVL was included as a covariate in the model to control for body size relationships with limb lengths. A caveat for this analysis is that because we lacked data on relatedness among wild toads, we exclude familial effects from the model. Thus sib-ships among common garden toads were ignored.

To assess familial similarity in limb morphology in a formal quantitative genetics framework, we also ran an ‘animal model’ using ASREML software (VSN International, Hemel Hempstead, UK) [57]. When pedigree information is available (as is the case for our common garden offspring), animal models can be used to estimate the genetic underpinning of phenotypic variation [57]. Because most individuals were measured on more than one occasion, we were also able to estimate the ontogenetic repeatability of relative limb lengths. We incorporated offspring ID and parental ID as random effects in the animal model and included SVL as a covariate to correct for body size and age. Although our sample was adequate to detect heritabilities, it was too small to calculate genetic correlations among traits [57]. All other analyses were performed using JMP 11 software (SAS Institute, Cary, NC).

## 3. Results

### 3.1. Sexual dimorphism varies with time since colonization in field-caught toads

**Figure 1.**
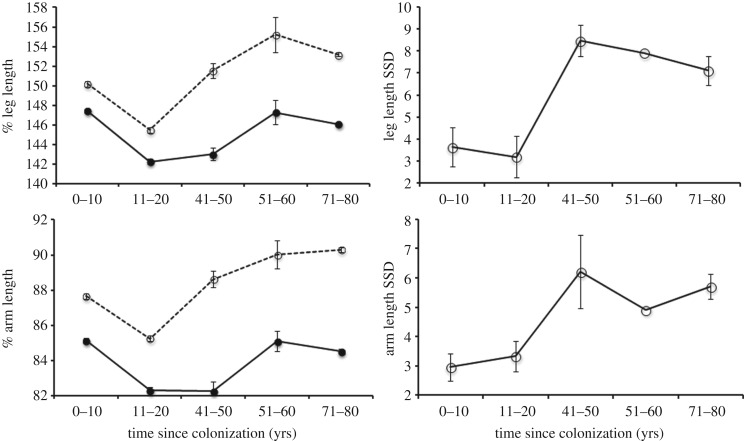
Comparisons of relative leg (top left) and arm (bottom left panel) lengths of 3094 wild-caught male and female cane toads from locations with different colonization times. Males are represented by open symbols and dashed lines, females by closed symbols and solid lines. Relative limb length values are expressed as a percentage of body length. Sexual dimorphism indices for each limb (calculated as the mean male value minus the mean female value) are presented in the corresponding right-hand panels. Error bars represent 1 standard error from the mean.

Relative limb lengths of wild-caught toads changed in different patterns with TSC in males versus females. Interactions between sex and TSC were highly significant both for arm length and leg length (table 1 and figure 1). Both sexes exhibited a ‘U’-shaped' (curvilinear) pattern with TSC for both limbs (figure 1); relative limb lengths were lowest in populations of intermediate TSC (11–20 years) than in either range-core or invasion-vanguard populations. Males had longer limbs than females in every population, but the magnitude of dimorphism was lower in recently invaded areas (i.e. Northern Territory and Western Australia) than in areas that were colonized many decades ago (i.e. Hawai'i, Queensland). The highest values for relative limb lengths in females were seen in invasion-front populations, whereas the highest values for males were seen in long-established populations.

**Table 1.** Effect of sex and time since colonization (TSC) categories on relative arm and leg lengths of 3094 wild-caught cane toads from Hawai'i (*N* = 1018) and Australia (*N* = 2075). Statistically significant values (*p* < 0.05) are highlighted in italics.

trait	variable	d.f.	mean square	*F*-value	*p*-value
total arm length	SVL	1	241558.3	19738.4	*<0.0001*
	sex	1	1973.7	161.3	*<0.0001*
	TSC	4	1302.6	106.4	*<0.0001*
	sex × TSC	4	407.3	33.3	*<0.0001*
	error	3080	12.2		
total leg length	SVL	1	704776.5	21233.9	*<0.0001*
	sex	1	2961.7	89.2	*<0.0001*
	TSC	4	3534.1	106.5	*<0.0001*
	sex × TSC	4	760.6	22.9	*<0.0001*
	error	3080	33.2		

### 3.2. Sexual dimorphism varies with parental location among common garden toads

**Figure 2.**
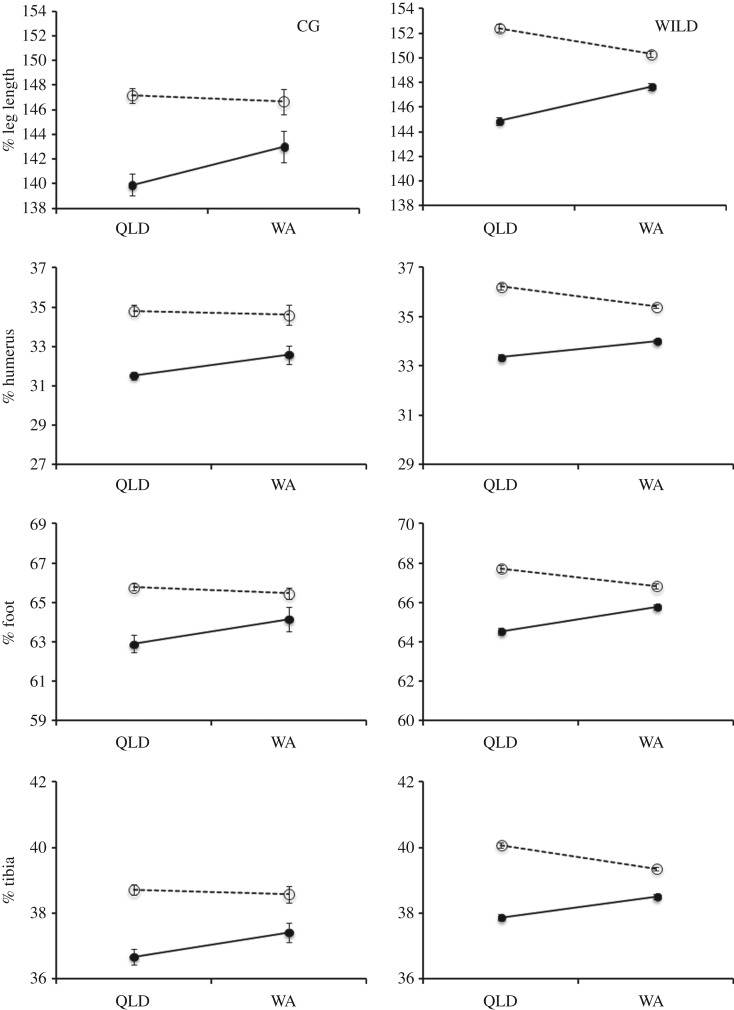
Relative limb lengths of cane toads from long-colonized versus invasion-front populations, as a function of whether the toads were wild-caught (right-hand panels) or captive-raised (left-hand panels). The left-hand panels show statistically significant interactions between sex and state for limb measures of male (open symbols, dashed line) and female (closed symbols, solid line) cane toads reared in a common environment. The parents of these toads originated either from long-established populations in QLD or from invasive populations in WA. Significant interactions for the same limb measures seen in wild male and female toads from the same locations are depicted in the right-hand panels. In each case, the limb length is expressed as a percentage of toad snout–vent length. Error bars represent one standard error from the mean.

Among the 184 captive-reared toads that reached maturity by the end of the study, the relative lengths of the humerus, foot, tibia and leg were affected by interactions between sex and the state of origin of their parents (QLD versus WA; table 2 and figure 2). These interactions broadly mirrored those seen in wild-caught toads from QLD versus WA (table 3 and figure 2). However, among wild toads, significant sex × state interactions were detected in all limb measures, whereas the same interactions were not statistically significant for some measures (hand, radioulna, arm and femur) among our smaller sample of common garden toads (table 2). When we assessed the relative lengths of the humerus, foot, tibia and leg in an analysis that combined data from the 184 common garden toads with the 1274 wild toads from the source populations, it verified the same significant interaction effect of state and sex found when each group was analysed separately (table 4). For all these measures, wild toads had significantly longer relative limb lengths than common garden toads. However, although source (wild versus common garden) was a significant main effect in all analyses, it did not appear in any significant interactions (table 4). Hence, wild-caught toads and those reared in captivity exhibited the same underlying pattern of decreased sexual dimorphism in relative limb lengths in invasive (WA) versus established (QLD) populations. This further supports the genetic basis of the patterns.

**Table 2.** ANOVA results show the effects of sex and state on limb sizes of 184 cane toads reared under common garden conditions. Thirty full-sib families produced offspring that reached maturity during the study and could be sexed based on secondary sexual characteristics. The parents of 17 families originated from long-established populations (Queensland) and the parents of 13 families originated from invasion-front populations (Western Australia). Only the final set of morphological measurements taken from each individual were included in this analysis. Snout-to-vent length (SVL) was included as a covariate in the model to adjust trait measures for body size. Family (nested within state) was included as a random effect in the model. Statistically significant values (*p* < 0.05) are highlighted in italics.

	SVL		sex		state		sex × state	
Trait	*F*	*P*	*F*	*P*	*F*	*P*	*F*	*P*
hand	260.8	*<0.0001*	5.1	*0.0255*	5.9	*0.0225*	1.5	0.2255
radioulna	430.8	*<0.0001*	68.9	*<0.0001*	2.0	0.1647	0.4	0.5380
humerus	466.3	*<0.0001*	94.5	*<0.0001*	1.0	0.3167	7.3	*0.0075*
total arm	624.0	*<0.0001*	78.7	*<0.0001*	1.2	0.2804	1.1	0.2996
foot	557.5	*<0.0001*	14.5	*0.0002*	0.4	0.5495	4.7	*0.0315*
tibia	783.1	*<0.0001*	42.0	*<0.0001*	0.6	0.4666	4.8	*0.0300*
femur	422.4	*<0.0001*	21.8	*<0.0001*	1.0	0.3316	3.6	0.0587
total leg	717.1	*<0.0001*	29.2	*<0.0001*	0.7	0.4020	5.7	*0.0177*

**Table 3.** ANOVA results show the effects of sex and state on limb sizes of 1274 wild-caught cane toads from long-established populations in Queensland (*N* = 467) and invasion-front populations in Western Australia (*N* = 807). Statistically significant values (*p* < 0.05) are highlighted in italics.

	SVL		sex		state		sex × state	
trait	*F*	*p*	*F*	*p*	*F*	*p*	*F*	*p*
hand	3082.7	*<0.0001*	32.1	*<0.0001*	39.1	*<0.0001*	26.1	*<0.0001*
radioulna	4853.4	*<0.0001*	291.5	*<0.0001*	8.4	*0.0037*	61.6	*<0.0001*
humerus	4607.6	*<0.0001*	413.4	*<0.0001*	1.6	0.2087	51.4	*<0.0001*
total arm	6714.4	*<0.0001*	357.5	*<0.0001*	17.1	*<0.0001*	73.2	*<0.0001*
foot	5127.2	*<0.0001*	90.6	*<0.0001*	24.8	*<0.0001*	42.5	*<0.0001*
tibia	8189.1	*<0.0001*	260.7	*<0.0001*	11.1	*0.0009*	71.5	*<0.0001*
femur	5025.2	*<0.0001*	119.5	*<0.0001*	12.4	*0.0004*	36.4	*<0.0001*
total leg	7615.8	*<0.0001*	174.7	*<0.0001*	22.8	*<0.0001*	61.4	*<0.0001*

**Table 4.** Effects of sex, state and source (wild versus common garden) on limb sizes of 1274 wild and 184 captive-reared toads from long-established populations in Queensland and invasion-front populations in West Australia. The significant main effect of source in all cases indicates that wild toads had relatively longer limbs than captive-reared toads. The significant shift in sexual dimorphism of limb lengths between states (indicated by significant sex × state interactions) does not differ between wild and common garden toads (all three-way interactions n.s.). Statistically significant values (*p* < 0.05) are highlighted in italics.

trait	effect	d.f.	*F*	*p*
total leg length	SVL	1,1447	8339.8	*<0.0001*
	state	1,1447	9.3	*0.0023*
	sex	1,1447	94.2	*<0.0001*
	state × sex	1,1447	21.9	*<0.0001*
	source	1,1447	182.3	*<0.0001*
	state × source	1,1447	0.23	0.6351
	sex × source	1,1447	0.5	0.4884
	state × sex × source	1,1447	0.4	0.5403
humerus	SVL	1,1449	5086.1	*<0.0001*
	state	1,1449	3.5	0.0628
	sex	1,1449	243.8	*<0.0001*
	state × sex	1,1449	21.9	*<0.0001*
	source	1,1449	114.5	*<0.0001*
	state × source	1,1449	0.7	0.4204
	sex × source	1,1449	2.3	0.1277
	state × sex × source	1,1449	0.04	0.8440
tibia	SVL	1,1449	9008.1	*<0.0001*
	state	1,1449	5.6	*0.0180*
	sex	1,1449	136.7	*<0.0001*
	state × sex	1,1449	23.4	*<0.0001*
	source	1,1449	167.1	*<0.0001*
	state × source	1,1449	0.01	0.9194
	sex × source	1,1449	0.3	0.5648
	state × sex × source	1,1449	0.7	0.3887
foot	SVL	1,1447	5697.3	*<0.0001*
	state	1,1447	6.9	*0.0087*
	sex	1,1447	47.7	*<0.0001*
	state × sex	1,1447	14.7	*0.0001*
	source	1,1447	121.7	*<0.0001*
	state × source	1,1447	1.1	0.2939
	sex × source	1,1447	0.2	0.6916
	state × sex × source	1,1447	0.4	0.5483

### 3.3. Estimates of heritability

Our estimates of heritability for relative limb measures (table 5) ranged from 0.02 ± 0.021 (femur) to 0.17 ± 0.043 (hand). These values suggest that most measures of limb morphology have a genetic component and hence are capable of responding to selection. The heritability estimates for femur and humerus, however, were low, and the confidence limits around them encompassed 0. These two traits may be less likely to respond to selection. We were able to measure limb components with high repeatability (table 5). Tibia length was the most highly repeatable measurement (0.99), and hand was the least repeatable (0.80). As toads grew larger, relative lengths of their limbs were moderately stable; ontogenetic repeatability ranged from 0.19 (foot) to 0.39 (tibia).

**Table 5.** Estimates of heritability, ontogenetic repeatability (of measures made at different ages on the same animals over a long period) and measurement repeatability (of successive measures taken on the same animal over a brief period) of limb measurements of cane toads reared in a common environment. Heritability and ontogenetic repeatability estimates were calculated from data on 550 individual toads (489 offspring, 61 parents). Measurement repeatability was calculated from a sample of five toads from which triplicate measures were made on a single occasion.

trait	heritability	ontogenetic repeatability	measurement repeatability
hand	0.17 ± 0.043	0.34 ± 0.033	0.80 ± 0.203
radioulna	0.17 ± 0.052	0.35 ± 0.033	0.91 ± 0.137
humerus	0.03 ± 0.024	0.33 ± 0.032	0.89 ± 0.147
total arm	0.13 ± 0.046	0.29 ± 0.033	0.92 ± 0.122
foot	0.11 ± 0.034	0.19 ± 0.033	0.97 ± 0.092
tibia	0.11 ± 0.044	0.39 ± 0.032	0.99 ± 0.025
femur	0.02 ± 0.021	0.23 ± 0.033	0.93 ± 0.101
total leg	0.10 ± 0.037	0.23 ± 0.033	0.98 ± 0.043

## 4. Discussion

The cane toad invasion of Australia has been accompanied by rapid changes to skeletal morphology. Among wild toads, relative leg lengths (ancestrally high) have decreased in the course of the invasion across Australia, and then increased again in invasion-vanguard populations. In contrast, sexual dimorphism in relative limb lengths exhibits a simpler monotonic decline: toads in ancestral (range-core) populations are highly dimorphic, whereas toads in invasion-front populations show little sex-based divergence in limb lengths. Relative limb length exhibits significant heritability, and captive-raised toads show similar patterns of morphology as their wild-caught parental populations, suggesting that these morphological shifts may represent evolved changes rather than (or as well as) developmentally plastic responses to different environments.

First, what processes have driven the curvilinear pattern of changes in relative limb length during the toads' Australian invasion? Annual rates of range expansion increased substantially over this period (from 10 to 15 km per annum to more than 60 km per annum [30]), potentially placing major stresses on a body plan that is poorly suited to continuous long-distance travel [43,58]. Long limbs provide high propulsive power for leaping, an effective tactic to evade an oncoming predator [59–62] but may be poorly suited to long periods of continuous slow dispersal over irregular terrain. Cane toads are capable of multiple locomotor modes, and small frequent hops (‘bounding’) may be more energetically efficient at traversing long distances than is a reliance on large single hops (‘leaping’ [63]). In addition, shorter hops may reduce biomechanical stresses on the toad's body. Long legs in invasion-front cane toads are associated with a high incidence of spinal arthritis [43,58]. Hence, natural selection in the course of the toad's long march across tropical Australia may have favoured individuals with shorter-than-average arms and legs that moved by bounding rather than by leaping. The arms play a major functional role in bounding [63–65], consistent with the shifts seen in both forelimbs and hindlimbs.

Why has this process reversed in populations close to the invasion front, with longer-limbed individuals (of both sexes) in these western sites? This reversal may be due to spatial sorting rather than natural selection. Even if selection favours animals with shorter limbs, the rate of dispersal is highest for long-legged toads (based on radio-tracking [30]). Thus, alleles for longer legs accumulate in the vanguard of the invasion, regardless of whether or not they enhance fitness of their bearers [2]. The result is that leg length decreases over the course of the invasion, but with a reversal close to the invasion front where spatial sorting overrides natural selection. Alternatively (or additionally), selective advantages that accrue to individuals in the invasion vanguard (more food, owing to lower densities of conspecifics) may favour maximal dispersal rates (and thus, longer legs) in this phase of the invasion. Faster dispersal would not confer the same fitness benefits in longer-colonized areas, because it would not enable individuals to reach low-density populations. Our data thus extend and clarify a previous report of longer hindlimbs in toads close to the invasion front [30]. Our extensive sampling reveals a more complex scenario, with invasion driving a reduction in relative limb length, but reversing to a rapid increase in leg (and arm) length near the invasion front.

In contrast to the curvilinear trends in relative limb lengths with TSC, the magnitude of sexual dimorphism in limb dimensions showed a rapid decline in populations colonized between 40 and 20 years ago (figure 1). Consistent with their greater limb lengths, male cane toads can travel faster than females (C. M. Hudson 2016, unpublished data from locomotor trials; see [33] for a similar sex difference in agility). All else being equal then, the evolution of more rapid dispersal during the toad invasion would have resulted in males substantially out-pacing females, thereby reaping the benefits of enhanced food availability at the invasion front [16], but at the cost of a highly skewed operational sex ratio (or in the extreme, a lack of females). The high degree of sex-based divergence in relative limb length in cane toads from range-core populations may result from sexual selection; previous studies have documented an association between limb muscle mass and reproductive success in male cane toads in the field [45,46]. Hence, selection may have favoured larger and/or more muscular limbs in male toads than females in ancestral populations; this dimorphic condition is widespread among anurans in general, including bufonids [47]. As soon as toads began dispersing westwards from Queensland, however (and especially, when that rate of dispersal accelerated [66]), novel forms of selection could have come into play, reshaping the ancestral cane toad body plan. First, low-density populations may render male–male competition less important at the invasion front (unless that trend is opposed by a shift in the operational sex ratio) and second, limb lengths affect not only potential dispersal rates, but also the energy and wear-and-tear associated with long-distance travel [43]. We cannot tease apart the relative importance of those two processes—sexual selection and natural selection—in driving cane toads towards sexual monomorphism, nor can we convincingly distinguish the impact of spatial sorting from selection. Nonetheless, our data strongly support the *a priori* prediction that a biological invasion can impose novel evolutionary forces that reduce the degree of sexual dimorphism in ancestral (range-core) populations.

We base our interpretative scenarios on adaptive mechanisms shifting morphological traits of toads over the course of their invasion. The similarity of traits between common garden-reared and wild toads and the non-zero heritability suggest that the trait changes have a genetic basis and are capable of responding to selection. Despite the plastic effects wrought by rearing differences (i.e. wild toads have significantly longer limbs than captive toads), the shift in limb dimorphism between established and invasive populations is strongly evident. Verification that the differences in traits have arisen through selection would require *Q*_st _– *F*_st_ analysis to compare shifts in the quantitative traits to concurrent shifts in neutral traits, however [53].

The heritabilities we calculated for relative limb length—around 10%—are lower than reported for morphological traits in many other species of animals [67]. Clearly, that leaves room also for significant environmental influences. Future work could usefully explore the sensitivity of toad limb lengths to larval conditions (as in [53]) and characterize the mating systems of toads at the invasion front compared with range-core populations. Regardless of uncertainty about causal mechanisms, however, our data document a substantial shift in morphology and sexual dimorphism within an invasive species, within the span of a single human lifetime.

## Data accessibility

All data used in this study can be accessed from the Dryad Digital Repository: http://dx.doi.org/10.5061/dryad.gk87k [68].

## References

[1] Moran EV, Alexander JM 2014 Evolutionary responses to global change: lessons from invasive species. Ecol. Lett. 17, 637–649. (doi:10.1111/ele.12262) 24612028 10.1111/ele.12262

[2] Shine R, Brown GP, Phillips BL 2011 An evolutionary process that assembles phenotypes through space rather than through time. Proc. Natl Acad. Sci. USA 108, 5708–5711. (doi:10.1073/pnas.1018989108) 21436040 10.1073/pnas.1018989108 PMC3078378

[3] Chuang A, Peterson CR 2015 Expanding population edges: theories, traits, and trade-offs. Glob. Change Biol. 22, 494–512. (doi:10.1111/gcb.13107) 10.1111/gcb.13107 26426311

[4] Rollins LA, Richardson MF, Shine R 2015 A genetic perspective on rapid evolution in cane toads (*Rhinella marina*). Mol. Ecol. 24, 2264–2276. (doi:10.1111/mec.13184) 25894012 10.1111/mec.13184

[5] Andersson M 1994 Sexual selection. Princeton, NJ: Princeton University Press.

[6] Travis JMJ, Dytham C 2002 Dispersal evolution during invasions. Evol. Ecol. Res. 4, 1119–1129.

[7] Simmons AD, Thomas CD 2004 Changes in dispersal during species’ range expansions. Am. Nat. 164, 378–395. (doi:10.1086/423430) 15478092 10.1086/423430

[8] Phillips BL, Brown GP, Greenlees M, Webb JK, Shine R 2007 Rapid expansion of the cane toad (*Bufo marinus*) invasion front in tropical Australia. Austral. Ecol. 32, 169–176. (doi:10.1111/j.1442-9993.2007.01664.x)

[9] Phillips BL, Brown GP, Travis JMJ, Shine R 2008 Reid's paradox revisited: the evolution of dispersal kernels during range expansion. Am. Nat. 172, S34–S48. (doi:10.1086/588255) 18554142 10.1086/588255

[10] Ronce O, Clobert J 2012 Dispersal syndromes. In Dispersal ecology and evolution (eds Clobert J, Baguette MG, Benton TG, Bullock JM), pp. 119–138. Oxford, UK: Oxford University Press.

[11] Cwynar LC, MacDonald GM 1987 Geographical variation of lodgepole pine in relation to population history. Am. Nat. 129, 463–469. (doi:10.1086/284651)

[12] Forsman A, Merilä J, Ebenhard T 2011 Phenotypic evolution of dispersal-enhancing traits in insular voles. Proc. R. Soc. B. 278, 225–232. (doi:10.1098/rspb.2010.1325) 10.1098/rspb.2010.1325 PMC3013397 20685710

[13] Berthouly-Salazar C, van Rensburg BJ, Le Roux JJ, van Vuuren BJ, Hui C 2012 Spatial sorting drives morphological variation in the invasive bird, *Acridotheris tristis*. PLoS ONE 7, e38145 (doi:10.1371/journal.pone.0038145) 22693591 10.1371/journal.pone.0038145 PMC3364963

[14] Therry L, Nilsson-Oertman V, Bonte D, Stoks R 2014 Rapid evolution of larval life history, adult immune function and flight muscles in a poleward-moving damselfly. J. Evol. Biol. 27, 141–152. (doi:10.1111/jeb.12281) 24313892 10.1111/jeb.12281

[15] Bitton PP, Graham BA 2015 Change in wing morphology of the European starling during and after colonization of North America. J. Zool. 295, 254–260. (doi:10.1111/jzo.12200)

[16] Brown GP, Kelehear C, Shine R 2013 The early toad gets the worm: cane toads at an invasion front benefit from higher prey availability. J. Anim. Ecol. 82, 854–862. (doi: 10.1111/1365-2656.12048) 23360501 10.1111/1365-2656.12048

[17] Miller TEX, Shaw AK, Inouye BD, Neubert MG 2011 Sex-biased dispersal and the speed of two-sex invasions. Am. Nat. 177, 549–561. (doi:10.1086/659628) 21508603 10.1086/659628

[18] Arnqvist G, Rowe L 2005 Sexual conflict. Princeton, NJ: Princeton University Press.

[19] Pearson D, Shine R, Williams A 2002 Geographic variation in sexual size dimorphism within a single snake species (*Morelia spilota*, Pythonidae). Oecologia 131, 418–426. (doi:10.1007/s00442-002-0917-5) 10.1007/s00442-002-0917-5 28547714

[20] Slip DJ, Shine R 1988 The reproductive biology and mating system of diamond pythons, *Morelia spilota* (Serpentes, Boidae). Herpetologica 44, 396–404.

[21] Kokko H, Rankin DJ 2006 Lonely hearts or sex in the city? Density-dependent effects in mating systems. Phil. Trans. R. Soc. B 361, 319–334. (doi:10.1098/rstb.2005.1784) 16612890 10.1098/rstb.2005.1784 PMC1569612

[22] Martin OY, Hosken DJ 2003 The evolution of reproductive isolation through sexual conflict. Nature 423, 979–982. (doi:10.1038/nature01752) 12827200 10.1038/nature01752

[23] Carranza J, Pérez-Barbería FJ 2007 Sexual selection and senescence: male size-dimorphic ungulates evolved relatively smaller molars than females. Am. Nat. 170, 370–380. (doi:10.1086/519852) 17879188 10.1086/519852

[24] Bonduriansky R, Maklakov A, Zajitschek F, Brooks R 2008 Sexual selection, sexual conflict and the evolution of ageing and life span. Funct. Ecol. 22, 443–453. (doi:10.1111/j.1365-2435.2008.01417.x)

[25] Travers LM, Garcia-Gonzalez F, Simmons LW 2015 Live fast die young life history in females: evolutionary trade-off between early life mating and lifespan in female *Drosophila melanogaster*. Sci. Rep. 5, 15469 (doi:10.1038/srep15469) 26482533 10.1038/srep15469 PMC4612512

[26] Shine R 1979 Sexual selection and sexual dimorphism in the Amphibia. Copeia 1979, 297–306. (doi:10.2307/1443418)

[27] Hudson CM, Fu J 2013 Male-biased sexual size dimorphism, resource defense polygyny, and multiple paternity in the Emei moustache toad (*Leptobrachium boringii*). PLoS ONE 8, e67502 (doi:10.1371/journal.pone.0067502) 23840725 10.1371/journal.pone.0067502 PMC3696078

[28] Chapman T, Arnqvist G, Bangham J, Rowe L 2003 Sexual conflict. Trends Ecol. Evol. 18, 41–47. (doi:10.1016/S0169-5347(02)00004-6)

[29] Freeland WJ 1986 Populations of cane toad *Bufo marinus* in relation to time since colonization. Aust. Wildl. Res. 13, 321–330. (doi:10.1071/WR9860321)

[30] Phillips BL, Brown GP, Webb JK, Shine R 2006 Invasion and the evolution of speed in toads. Nature 439, 803 (doi:10.1038/439803a) 16482148 10.1038/439803a

[31] Hudson CM, McCurry MR, Lundgren P, McHenry CR, Shine R 2016 Constructing an invasion machine: the rapid evolution of a dispersal-enhancing phenotype during the cane toad invasion of Australia. PLoS ONE 11, e0156950 (doi:10.1371/journal.pone.0156950) 27658247 10.1371/journal.pone.0156950 PMC5033235

[32] Llewelyn J, Phillips BL, Alford RA, Schwarzkopf L, Shine R 2010 Locomotor performance in an invasive species: cane toads from the invasion front have greater endurance, but not speed, compared to conspecifics from a long colonised area. Oecologia 162, 343–348. (doi:10.1007/s00442-009-1471-1) 19841946 10.1007/s00442-009-1471-1

[33] Hudson CM, Brown GP, Shine R 2016 Athletic anurans: the impact of morphology, ecology and evolution on climbing ability in invasive cane toads. Biol. J. Linn. Soc. (doi:10.1111/bij.12827)

[34] Phillips BL, Brown GP, Shine R 2010 Evolutionarily accelerated invasions: the rate of dispersal evolves upwards during the range advance of cane toads. J. Evol. Biol. 23, 2595–2601. (doi:10.1111/j.1420-9101.2010.02118.x) 20939838 10.1111/j.1420-9101.2010.02118.x

[35] Alford RA, Brown GP, Schwarzkopf L, Phillips BL, Shine R 2009 Comparisons through time and space suggest rapid evolution of dispersal behaviour in an invasive species. Wildl. Res. 36, 23–28. (doi:10.1071/WR08021)

[36] Lindström T, Brown GP, Sisson S, Phillips BL, Shine R 2013 Rapid shifts in dispersal behaviour on an expanding range edge. Proc. Natl Acad. Sci. USA 110, 13 452–13 456. (doi:10.1073/pnas.1303157110) 10.1073/pnas.1303157110 PMC3746873 23898175

[37] Brown GP, Phillips BL, Shine R 2014 The straight and narrow path: the evolution of straight-line dispersal at a cane-toad invasion front. Proc. R. Soc. B. 281, 20141385 (doi:10.1098/rspb.2014.1385) 10.1098/rspb.2014.1385 PMC4213614 25297862

[38] Tingley R, Greenlees MJ, Shine R 2012 Hydric balance and locomotor performance of an anuran (*Rhinella marina*) invading the Australian arid zone. Oikos 121, 1959–1965. (doi:10.1111/j.1600-0706.2012.20422.x)

[39] McCann S, Greenlees MJ, Newell D, Shine R 2014 Rapid acclimation to cold allows the cane toad to invade montane areas within its Australian range. Funct. Ecol. 28, 1166–1174. (doi:10.1111/1365-2435.12255)

[40] Brown GP, Phillips BL, Dubey S, Shine R 2015 Invader immunology: invasion history alters immune system function in cane toads (*Rhinella marina*) in tropical Australia. Ecol. Lett. 18, 57–65. (doi:10.1111/ele.12390) 25399668 10.1111/ele.12390

[41] Phillips BL, Brown GP, Shine R 2010 Life-history evolution in range-shifting populations. Ecology 91, 1617–1627. (doi:10.1890/09-0910.1) 20583704 10.1890/09-0910.1

[42] Hudson CM, Phillips BL, Brown GP, Shine R 2015 Virgins in the vanguard: low reproductive frequency in invasion front cane toads. Biol. J. Linn. Soc. 116, 743–747. (doi:10.1111/bij.12618)

[43] Brown GP, Shilton C, Phillips BL, Shine R 2007 Invasion, stress, and spinal arthritis in cane toads. Proc. Natl Acad. Sci. USA 104, 17 698–17 700. (doi:10.1073/pnas.0705057104) 10.1073/pnas.0705057104 PMC2077021 17951431

[44] Ducatez S, Crossland M, Shine R 2016 Differences in developmental strategies between long-settled and invasion-front populations of the cane toad in Australia. J. Evol. Biol. 29, 335–343. (doi:10.1111/jeb.12785) 26549779 10.1111/jeb.12785

[45] Lee JC 2001 Evolution of secondary sexual dimorphism in the toad, *Bufo marinus*. Copeia 2001, 928–935. (doi:10.1643/0045-8511(2001)001[0928:EOASSD]2.0.CO;2)

[46] Lee JC, Corrales AD 2002 Sexual dimorphism in hind-limb muscle mass is associated with male reproductive success in *Bufo marinus*. J. Herpetol. 36, 502–505. (doi:10.2307/1566198)

[47] Wells KD 2007 The ecology and behaviour of amphibians. Chicago, IL: University of Chicago Press.

[48] Hagman M, Hayes RA, Capon RJ, Shine R 2009 Alarm cues experienced by cane toad tadpoles affect post-metamorphic morphology and chemical defences. Funct. Ecol. 23, 126–132. (doi:10.1111/j.1365-2435.2008.01470.x)

[49] Witjethunga U, Greenlees M, Shine R 2016 Living up to its name? The effect of salinity on development, growth and phenotype of the ‘marine’ toad (*Rhinella marina*). J. Comp. Physiol. B 186, 205–213. (doi:10.1007/s00360-015-0944-2) 26553545 10.1007/s00360-015-0944-2

[50] Relyea RA 2001 The lasting effects of adaptive plasticity: predator induced tadpoles become long-legged frogs. Ecology 82, 1947–1955. (doi:10.1890/0012-9658(2001)082[1947:TLEOAP]2.0.CO;2)

[51] Van Buskirk J, Saxer G 2001 Delayed costs of an induced defense in tadpoles? Morphology, hopping, and development rate at metamorphosis. Evolution 55, 821–829. (doi:10.1554/0014-3820(2001)055[0821:DCOAID]2.0.CO;2) 11392399 10.1554/0014-3820(2001)055[0821:dcoaid]2.0.co;2

[52] Relyea RA, Hoverman JT 2003 The impact of larval predators and competitors on the morphology and fitness of juvenile tree frogs. Oecologia 134, 596–604. (doi:10.1007/s00442-002-1161-8) 12647133 10.1007/s00442-002-1161-8

[53] Alho JS, Herczeg G, Laugen AT, Räsänen K, Laurila A, Merilä J 2011 Allen's rule revisited: quantitative genetics of extremity length in the common frog along a latitudinal gradient. J. Evol. Biol. 24, 59–70. (doi:10.1111/j.1420-9101.2010.02141.x) 20964781 10.1111/j.1420-9101.2010.02141.x

[54] Alford RA, Cohen MP, Crossland MR, Hearnden MN, Schwarzkopf L 1995 Population biology of *Bufo Marinus* in northern Australia. In Wetland research in the wet-dry tropics of Australia (ed. Finlayson M), pp. 173–181. Supervising Scientist Report No. 101. Canberra, Australia: Office of the Supervising Scientist.

[55] Phillips BL, Kelehear C, Pizzatto L, Brown GP, Barton D, Shine R 2010 Parasites and pathogens lag behind their host during periods of host range-advance. Ecology 91, 872–881. (doi:10.1890/09-0530.1) 20426344 10.1890/09-0530.1

[56] Nakagawa S, Schielzeth H 2010 Repeatability for Gaussian and non-Gaussian data: a practical guide for biologists. Biological Reviews 85, 935–956. 20569253 10.1111/j.1469-185X.2010.00141.x

[57] Wilson AJ, Reale D, Clements MN, Morrissey MM, Postma E, Walling CA, Kruuk LE, Nussey DH 2010 An ecologist's guide to the animal model. J. Anim. Ecol. 79, 13–26. (doi:10.1111/j.1365-2656.2009.01639.x) 20409158 10.1111/j.1365-2656.2009.01639.x

[58] Shilton CM, Brown GP, Benedict S, Shine R 2008 Spinal arthropathy associated with *Ochrobactrum anthropi* in free-ranging cane toads (*Chaunus* [*Bufo*] *marinus*) in Australia. Vet. Pathol. 45, 85–94. (doi:10.1354/vp.45-1-85) 18192584 10.1354/vp.45-1-85

[59] Gans C, Parsons TS 1966 On the origin of the jumping mechanism in frogs. Evolution 20, 92–99. (doi:10.2307/2406151) 10.1111/j.1558-5646.1966.tb03345.x 28564747

[60] Emerson SB 1978 Allometry and jumping in frogs: helping the twain to meet. Evolution 32, 551–564. (doi:10.2307/2407721) 10.1111/j.1558-5646.1978.tb04598.x 28567959

[61] Zug GR 1985 Anuran locomotion: fatigue and jumping performance. Herpetologica 41, 188–194.

[62] Essner RL Jr, Suffian DJ, Bishop PJ, Reilly SM 2010 Landing in basal frogs: evidence of saltational patterns in the evolution of anuran locomotion. Die Naturwissenschaften 97, 935–939. (doi:10.1007/s00114-010-0697-4) 20625697 10.1007/s00114-010-0697-4

[63] Reilly SM, Montuelle SJ, Schmidt A, Naylor E, Jorgensen ME, Halsey LG, Essner RL 2015 Conquering the world in leaps and bounds: hopping locomotion in toads is actually bounding. Funct. Ecol. 29, 1308–1316. (doi:10.1111/1365-2435.12414)

[64] Akella T, Gillis GB 2011 Hopping isn't always about the legs: forelimb muscle activity patterns during toad locomotion. J. Exp. Zool. 315A, 1–11. (doi:10.1002/jez.643) 10.1002/jez.643 20872875

[65] Griep S, Schilling N, Marshall P, Amling M, Hahme LM, Haas A 2013 Pectoral girdle movements and the role of the glenohumeral joint during landing in the toad, *Rhinella marina* (Linnaeus, 1758). Zoomorphology 132, 325–338. (doi:10.1007/s00435-013-0189-0)

[66] Urban MC, Phillips BL, Skelly DK, Shine R 2007 The cane toad's (*Chaunus* [*Bufo*] *marinus*) increasing ability to invade Australia is revealed by a dynamically updated range model. Proc. R. Soc. B 274, 1413–1419. (doi:10.1098/rspb.2007.0114) 10.1098/rspb.2007.0114 PMC2176198 17389221

[67] Mousseau TA, Roff DA 1987 Natural selection and the heritability of fitness components. Heredity 59, 181–197. (doi:10.1038/hdy.1987.113) 3316130 10.1038/hdy.1987.113

[68] Hudson CM, Brown GP, Shine R 2016 Data from: It is lonely at the front: contrasting evolutionary trajectories in male and female invaders. Dryad Digital Repository. (doi:10.5061/dryad.gk87k) 10.1098/rsos.160687 PMC5210690 28083108

